# Effect of antibiotic withdrawal in feed on chicken gut microbial dynamics, immunity, growth performance and prevalence of foodborne pathogens

**DOI:** 10.1371/journal.pone.0192450

**Published:** 2018-02-14

**Authors:** Sanjay Kumar, Chongxiao Chen, Nagaraju Indugu, Gabriela Orosco Werlang, Manpreet Singh, Woo Kyun Kim, Harshavardhan Thippareddi

**Affiliations:** 1 Department of Poultry Science, University of Georgia, Athens, GA, United States of America; 2 New Bolton Center, University of Pennsylvania, Kennett Square, PA, United States of America; 3 Preventive Veterinary Medicine Department, Federal University of Rio Grande do Sul, Brazil; University of Connecticut, UNITED STATES

## Abstract

Development of antibiotic resistance in foodborne pathogens, *Salmonella* spp. and *Campylobacter*, is a public health concern. Public demand to reduce the use of sub-therapeutic antibiotic growth promoters (AGP) in poultry feeding has resulted in greater adoption of antibiotic-free poultry production systems. There is a need to understand the effects of AGP removal from poultry feed on gut microbiota and its impact on prevalence of foodborne pathogens. The effect of antibiotic withdrawal from poultry feed on gut microbial community, host performance and immunity, and prevalence of *Salmonella* and *Campylobacter* was evaluated. Birds were raised on three phase diets (starter [d0-22], grower [d23-35] and finisher [d36-42]) with and without bacitracin dimethyl salicyclate (BMD). At early growth stage, bird performance was improved (P ≤ 0.05) with BMD treatment, whereas performance was better (P ≤ 0.05) in control group (no BMD in the feed) at the time of commercial processing. Acetate and butyrate production was affected (P ≤ 0.05) by age, whereas propionate production was affected (P ≤ 0.05) by both the treatment and age. The bacterial communities in the cecum were more diverse (P ≤ 0.001) and rich compared to the ileal communities, and they shifted in parallel to one another as the chicks matured. Differences in diversity and species richness were not observed (P > 0.05) between the BMD-fed and control groups. Comparing all ages, treatments and diets, the composition of cecal and ileal bacterial communities was different (P ≤ 0.001). Inclusion of BMD in the feed did not affect the bacterial phyla. However, predictable shift in the ileal and cecal bacterial population at lower taxonomic level was observed in control vs BMD-fed group. Cytokines gene expression (IL-10, IL-4, IFN-γ, beta-defensin, and TLR-4) was affected (P≤ 0.05) in the BMD-fed group at early stages of growth. The prevalence of foodborne pathogens, *Campylobacter* spp. and *Salmonella* spp. showed higher abundance in the ilea of BMD-fed chicks compared to control group. Overall, this study provided insight of the impact of AGP supplementation in the feed on gut microbial modulations, bird performance, host immunity and pathogen prevalence. This information can assist in designing alternative strategies to replace antibiotics in modern poultry production and for food safety.

## Introduction

Antibiotics have been used in the poultry industry in the United States and other countries, for more than five decades. Supplementation of antibiotics as sub-therapeutics improves bird feed efficiency and maintain the gut health, growth and development [1, 2]. In North America, antibiotic growth promoters (AGPs), commonly used in the poultry industry include: Avilamycin, Enramycin, Monensin, Penicillin, Virginamycin and Bacitracin methylene disalicylate (BMD) [3]. BMD is commonly used in the broiler diet for the prevention and control of necrotic enteritis, as well as improvement of weight gain and feed efficiency [4, 5]. Inclusion of antibiotics in poultry diet can also reduce the prevalence of enteric pathogens. Regardless of successful use of AGPs, the definitive mechanism underlying their growth promoting effect is still unresolved. With increasing concern over agricultural use of antibiotics as growth promoters (AGPs) and the emergence and dissemination of antibiotic resistance in foodborne pathogens, there is consumer pressure to eliminate the use of AGPs as feed additives in the U.S. Therefore, search for alternative strategies to replace antibiotics as a feed additive has gained interest in animal agriculture.

Avian gastrointestinal tract is much shorter compared to the mammalian gastrointestinal tract and the average transit time is less than 3.5 h [6]. This short transit time selects for the bacterial community with better adherence property and faster growth in the ileum and other proximal part of gut. On the other hand, passage time in the ceca is slow and thus, represents an ideal habitat for the bacterial community [7]. The gut microbial community is diverse and complex, and their interactions significantly affect the physiological, immunological and nutritional status of the host [8]. This complex interaction can have either beneficial or detrimental effect on the bird performance and health, depending on the structure and function of the gut microbial community. For instance, pathogen infection affects gut integrity and function [9] and poses a threat to the immune system [10]. Antimicrobial peptides (β-defensins) in the avian gut are important part of innate immune system that can destroy various enteric pathogens by disrupting their cell membranes. These initial interactions between gut microbial community and host innate immune system can lead to subsequent adaptive immune response, which can either be B-cell dependent or T-cell dependent. [7]. Therefore, gut community helps in supporting proper development and homeostasis of immune system [11]. Further, microbial community helps in excluding pathogenic taxa, fermenting complex polysaccharides and providing energy as volatile fatty acids to the host [7].

Although the gut microbial community is stable, it is affected by dietary changes, inclusion of feed additives, prebiotics, probiotics, pathogenic infections and antibiotic administration [7, 8, 12]. Bird age also has a significant effect on the microbial community, and greater diversity occurs at species level [13, 14]. While literature on the impact of AGPs in feed on microbial composition in the gut is extensive [3, 14–16], information on the effect of AGPs on the gut environment and on the establishment and development of the intestinal microbiome of chickens is lacking. This impairs our understanding on how these gut-targeted treatments interacts with the host, and how they might affect bird performance, and eventually bird health. A better understanding of poultry gut microbiome using high-throughput next-generation sequencing (HT-NGS) and their interactions with host will allow design of alternative nutritional strategies (to replace antibiotics in feed) and reduce risk of pathogen for better bird health and productivity.

In the present study, we combined high-throughput sequencing approach targeting on V3- V4 hypervariable region of 16S rRNA with quantitative-PCR of cytokine gene expression to elucidate the effect of antibiotic withdrawal on ileal and cecal microbial community, bird performance and host immunity from the day of hatch until commercial weight gain/ processing and concurrent prevalence of *Salmonella* and *Campylobacter* in the gut of broilers.

## Materials and methods

All bird management and research procedures were performed under animal use protocols approved by the Institutional Animal Care and Use committee, University of Georgia (A2016 04–017).

### Experimental design and sample collection

The experiment was performed using day 0 hatched male Cobb500 broilers (n = 240) obtained from Cobb-Vantress hatchery, Cleveland, GA. The birds were randomly assigned to two treatment (2 treatments x 6 reps x 20 birds/pen) groups. One treatment group was fed bacitracin methylene disalicylate (BMD-50, Zoetis, MI) supplemented diet, while the other group was fed BMD-free diet (control). All the birds were raised on three phase diets from d0 to 42. Starter diets were offered to the birds from day-old until d21 of age, Grower diets from d22-35 and finishing diets from d36-42. Diets were formulated to meet the nutrient requirement recommended by NRC (1994) for broilers (Table 1). Diets and water were provided *ad libitum*. At d0 (pre-treatment), and d7, 14, 22, 35 and 42 (post-treatment), 6 chicks were randomly selected from each group per time point and euthanized by cervical dislocation. For pathogen isolation, ceca and ilea (n = 6 for each group per time point) were aseptically collected from each bird and maintained on ice. For DNA and RNA extraction and VFA analysis, cecal and ileal samples were snap frozen in liquid nitrogen. On d0 and 7, some chicks yielded minimal or no cecal/ ileal content, resulting in inadequate DNA for processing. Feed and litter samples (n = 6) were also collected for each group at each time point.

**Table 1:** Nutrient composition of the basal starter, grower and finisher diets.

**Ingredients composition (% DM)**	**Starter 0–21**	**Grower 21–35**	**Finisher 35–42**
		
Corn	53.28	62.41	69.59
Soybean	34.50	28.30	21.49
Dicalcium Phosphate	1.75	1.19	1.51
Soybean oil	5.60	4.31	3.09
Limestone	1.43	1.51	1.12
Sand*	0.60	0.65	0.65
L-Lysine	0	0	0.04
DL-Methionine	0.28	0.10	0.05
Common Salt	0.30	0.30	0.30
Threonine	1.97	1.00	1.92
Mineral Premix	0.08	0.08	0.08
Amprolium	0.05	0.05	0.05
25-(OH) Vit D_3_	0.05	0	0
Premix Control	0.1	0.1	0.1
Premix Low dose	0	0	0
Premix High dose	0	0	0
**Chemical composition (% DM)**			
Crude protein	23	20	18
ME (Kcal/Kg)	3,200	3,200	3,200
Lysine	1.16	1.00	0.85
Methionine	0.60	0.40	0.32
Total sulfur amino acids	0.95	0.72	0.60

For treatment group, basal diets were supplemented with 0.05% BMD-50 replacing 0.05% of the sand.

### Bird performance, pH, and VFA analysis

To assess the effect of BMD withdrawal from diet on bird performance, following performance characteristics were measured: body weight (BW), body weight gain (BWG), feed intake (FI) and feed conversion ratio (FCR), for each growth period (d0 to 42) and for each group. For pH determination, 1.0 gram of cecal or ileal content was diluted with 9 mL of sterilized cold water. The suspension was thoroughly stomached (Neutec Group Inc., Farmingdale, NY, USA), mixed using a magnetic stirrer (Thermo Fisher Scientific, Waltham, MA, USA), and pH was measured using a glass electrode (Hanna Instruments Inc., Carrollton, TX, USA).

The VFA concentrations in the cecal and ileal contents (n = 4 from each treatment group for each time point) were determined using the method of Cottyn and Boucque [17]. The sample was diluted and centrifuged for 10 min at 10,000 *g* at 4°C. Fluid supernatant (5.0 mL) was mixed with 1.0 mL of a metaphosphoric acid: crotonic acid (internal standard) solution and stored overnight at -20°C. Samples were thawed and centrifuged for 10 min at 10,000 *g* at 4°C. The supernatant was analyzed for VFA using a gas chromatography (Shimadzu GC-2010 Plus; Shimadzu Corporation, Kyoto, Japan) with a flame ionization detector, a capillary column (ZebronTM ZB-FFAP; 30 m x 0.32 mm x 0.25 μm; Phenomenex Inc., Torrance, CA, USA), and an injection volume of 1.0 μL. Injection temperature was set at 250°C at a 20:1 split ratio, and oven temperature was set at 100°C for 2 min, increased at a rate of 8°C/min to 200°C and held for 1 min. Carrier gas (He) set at a flow rate of 1.69 mL/minute was used. The concentrations of VFAs were determined by a comparison of sample peak heights with those of authentic standards (Sigma-Aldrich, USA).

### DNA extraction, 16 S rRNA amplification and sequencing

The archived cecal and ileal samples were thawed and DNA was then extracted using a bead-beating method [2] followed by QIAmp DNA stool mini kits (Qiagen Inc., Valencia, CA, USA). For Feed and litter, samples were diluted with PBS buffer in the ratio of 1:5, centrifuged at 10,000 *g* for 10 min, the pellet was collected and used for DNA extraction. DNA was then quantified using a NanoDrop 2000 spectrophotometer (NanoDrop, Wilmington, DE, USA), and quality was checked using agarose gel (0.8%) electrophoresis. The V3-V4 hypervariable region of the 16S rRNA gene was amplified using forward primer 5’ TCGTCGGCAGCGTCAGATGTGTATAAGAGACAGCCTACGGGNGGCWGCAG 3’ and reverse primer 5’GTCTCGTGGGCTCGGAGATGTGTATAAGAGACAGGACTACHVGGGTATCTAATCC 3’ adapted from Klindworth et al. [18] using HiFi Hotstart Readymix PCR kits (KAPA Biosystems Inc., Wilmington, MA, USA). The 16S rRNA library was prepared using Nextera XT Index 1and 2 primers (Illumina Inc., Hayward, CA, USA), and the amplicons were bead purified using Agencourt AMpure XP beads (Beckman Coulter, Inc., Brea, CA, USA). The amplicons were sequenced at Georgia Genomic Facility, University of Georgia for Illumina MiSeq 300-bp paired-end sequencing.

### RNA isolation and quantitative real-time PCR

The cecum and ileum (n = 4 for each group per time point) were washed in PBS and placed in a 2 mL micro centrifuge tube with 1 mL of RNAlater (Qiagen Inc., Valencia, CA), and stored at -80°C until processed. RNA was extracted from 40–60 mg of intestinal tissue with QIAzol Lysis Reagent (Qiagen Inc., Valencia, CA, USA) and 1.5 mm zirconium beads followed by isopropanol precipitation of the aqueous phase. RNA was resuspended in DEPC-water and quantified using a NanoDrop 2000 spectrophotometer (NanoDrop, Wilmington, DE, USA). cDNA was synthesized from RNA samples using Quantitect Reverse Transcription kits (Qiagen Inc., Valencia, CA, USA) containing random hexamer primers according to the protocol provided by the manufacturer. Quantitative real-time polymerase chain reaction (qRT-PCR) was performed in duplicate reactions, including nuclease free water, the forward and reverse primers of each gene (interferon- γ: IFN-γ, tumor necrosis factor– α: TNF-α, beta-defensin-4, interleukin-4: IL-4, interleukin-10: IL-10, and toll-like receptor-4: TLR-4), cDNA and SYBR green (Thermo Fisher Scientific, Waltham, MA, USA) as a detector on a StepOne Plus Real-Time PCR Detection System (Applied Biosystem, Waltham, MA, USA). The reaction program consisted of following cycle profile: one cycle of 95 °C for 10 min and 40 cycles at 95 °C for 15 s and at 72 °C for 15 s. Pairs of the primers for the cytokines and housekeeping gene, Glyceraldehyde 3-phosphate dehydrogenase (GAPDH), and annealing temperature used for qRT-PCR assays are presented in Table 2.

**Table 2:** Real-time quantitative RT-PCR primers for the chicken pro and anti-inflammatory cytokines.

RNA target^1^	Forward Primer	Reverse Primer	Annealing temperature	Product Size	Reference Sequence
**GAPDH**	5”CCTCTCTGGCAAAGTCCAAG3”	5”GGTCACGCTCCTGGAAGATA3”	56°C	176	NM_204305.1
**IL-10**	5”AGCAGATCAAGGAGACGTTC3”	5”ATCAGCAGGTACTCCTCGAT3”	58°C	103	NM_001004414.2
**IL-4**	5”TGTGCCCACGCTGTGCTTACA3”	5”CTTGTGGCAGTGCTGGCTCTCC3”	62°C	193	NM_001007079.1
**IFN-gamma**	5”CTGAAGAACTGGACAGAGAG3”	5”CACCAGCTTCTGTAAGATGC3”	58°C	264	NM_205149.1
**TLR-4**	5”TCCGTGCCTGGAGGTAAGT3”	TGCCTTGGTAACAGCCTTGA	57°C	155	NM_001030693
**AvBD4**	5”TTCTCTGCAGTGACAGGATTTCC3”	5”AAGCCCACAGCTCCATGAACT3”	59°C	101	NM_001001610.2

^1^IL = interleukin, IFN = interferon, TLR = Toll-like receptor, AvBD4 = Avian beta-defensin 4

Standard curve analysis was used to check the efficiency of each primer set. Template cDNA was diluted into 10-fold series and slope of standard curve was used to estimate the PCR amplification efficiency. The PCR efficiency of the primers was between 90.6–99.4% (−3.57 ≥ slope ≥ −3.33). A dissociation curve was run for each primer reaction to confirm the amplification of a single product. Data were generated using ΔΔCt method by normalizing the expression of a target gene to a housekeeping gene, GAPDH, and the values were reported as a fold change of the expression of the target genes in the treatment group compared to the control group across all ages.

### Enteric pathogen isolation

For *Salmonella* isolation, 0.5- and 0.1-mL of enriched culture were transferred to 10 mL tetrathionate broth (TTB; Becton, Dickinson and Company, Franklin Lakes, NJ, USA) and Rappaport-Vassiliadis (RV; Becton, Dickinson and Company, Franklin Lakes, NJ, USA) broth and incubated for 24 h at 42±1°C. After selective enrichment, a loopful of TT or RV broth culture was streaked for isolation onto xylose lysine tergitol 4 (Becton, Dickinson and Company, Franklin Lakes, NJ, USA) agar, and incubated overnight at 37±1°C. Four presumptive, well isolated *Salmonella* colonies on each XLT4 plate were picked and inoculated onto a triple sugar iron slant (Becton, Dickinson and Company, Franklin Lakes, NJ, USA) and incubated for 24 h at 35±1°C.

Isolation and culturing of *Campylobacter* was done under microaerophilic conditions (AnaeroPak system; Campygen^TM^, Thermo Fisher Scientific, Waltham, MA, USA). Cecal and ileal contents (1 mL) were enriched in Bolton broth (Becton, Dickinson and Company, Franklin Lakes, NJ, USA) and incubated at 42°C overnight. The overnight enrichment broth was streaked for isolation onto Campy-cefex plates (Acumedia Manufacturer Inc., Lansing, MI, USA) and incubated for 48 h at 42°C, the plates were examined for typical colonies of *Campylobacter*.

### Bioinformatics analysis

Sequencing data analyzed using the Quantitative Insights Into Microbial Ecology (QIIME) pipeline (v.1.8.0; [19]). The paired-end reads were stitched together and joined reads were demultiplexed and quality filtered. A de novo operational taxonomic units (OTUs) were formed at 97% similarity using UCLUST [20]. Representative sequences from each OTU were aligned to 16S reference sequences with PyNAST [21] and used to infer a phylogenetic tree with FastTree [22]. Taxonomic assignments within the GreenGenes taxonomy [12/10 release] [23] were generated using uclust consensus taxonomy assigner. The OTU tables were rarefied at 13,265 sequences per sample for alpha and beta diversity. Analyses of community similarity (β-diversity) were performed by calculating pairwise distance using phylogenetic metric UniFrac [24]. Three measures of alpha diversity were computed including Good’s Coverage, an indicator of sequencing depth, Shannon diversity, an indicator of evenness in community structure, and richness, the number of OTUs observed. OTU abundances were normalized to the total number of reads in each sample (relative abundance), to test the differences in taxon abundances. Phyla/genera appearing in at least 75% of samples were considered.

### Statistical analysis

All the statistical analyses were performed in R software version (R Core Team, 2013). Wilcoxon signed rank test was used for the comparison between BMD-fed (n = 6) and control group (n = 6) at each time point (d0, 7, 14, 22, 35 and 42) for cecal, ileal, litter and feed samples using Shannon diversity and Observed species matrices. A non-parametric per mutational multivariate ANOVA test [25], implemented in the vegan package for R [26, 27], was used to test the effects of treatment, time and diets on overall community composition on the weighted and un-weighted UniFrac distances. A generalized linear mixed-effects model (glmer) was constructed with the lme4 package for R, to test the effects of treatment, time and diets on each taxa abundance [28].

The VFA data and gene expression data were subjected to 2-way ANOVA by using the GLM procedure of the SAS [29]. The model included the main effect of treatments, age and their interaction. The pen was considered as the experimental unit. The means showing significant (P≤0.05) treatment differences in the ANOVA were then compared using Tukey’s test. All data were tested for normality and homogeneity of variances, using the UNIVARIATE procedure and Bartlett test of SAS system [29], respectively.

## Results

### Bird performance

BMD diet enhanced (P ≤ 0.05) early weight gain in the bird (Table 3). In contrast, at d36-42, improvement (P ≤ 0.05) in BW, BWG and FI was observed in the control (BMD-free diet) group. These results indicate that although supplementing an antibiotic in the feed improves bird performance during the early growth period, this advantage was lost during the later stages of growth and at the time of commercial processing.

**Table 3:** Bird performance (BW. BWG, FI and FCR) for BMD-fed and no BMD-fed group birds at different ages.

Diet	Starter												Grower				Finisher			
Day	0–7				8–14				15–22				23–35				36–42			
Parameters	BW (g)	BWG (g)	FI (g)	FCR(g)	BW (g)	BWG (g)	FI (g)	FCR(g)	BW (g)	BWG (g)	FI (g)	FCR(g)	BW (g)	BWG (g)	FI (g)	FCR(g)	BW (g)	BWG (g)	FI (g)	FCR(g)
**BMD group**	142.83^a^	99.61^a^	125.00^a^	1.26^a^	371.23^a^	229.38^a^	281.19^a^	1.23^a^	870.46^a^	503.80^a^	639.78^a^	1.27^a^	2027.25^a^	1171.85^a^	1879.11^a^	1.60^a^	2451.93^a^	494.45^a^	1038.54^a^	2.03^a^
**No-BMD group**	146.20^a^	103.52^a^	129.86^a^	1.26^a^	356.81^b^	211.59^b^	273.63^a^	1.29^b^	798.64^b^	444.01^a^	588.73^b^	1.35^a^	2007.52^a^	1265.26^a^	1870.36^a^	1.49^a^	2747.90^b^	648.87^b^	1225.44^b^	1.92^a^
**SEM**	1.688	1.637	1.768	0.017	4.158	3.256	4.820	0.017	21.339	21.551	15.295	0.048	45.854	42.304	46.211	0.036	82.388	39.297	42.819	0.060
*P* value	0.1895	0.1225	0.0835	0.9938	0.038	0.0032	0.3075	0.0206	0.049	0.1027	0.0464	0.3391	0.7803	0.1988	0.9076	0.0663	0.0315	0.0197	0.0115	0.3042

BW: body weight/bird; BWG: body weight gain/bird; FI: feed intake/bird; FCR: feed conversion ratio/bird; SEM: standard error of mean (n = 6)

^a,b^means in the same column with no common superscript letters differ significantly (P ≤ 0.05)

Acetate production was higher, followed by butyrate and propionate, post treatment (d7-42). In the cecum, difference (P ≤ 0.001) in acetate and butyrate production was observed as bird matured, and treatment (BMD feeding) effect was not observed. On the other hand, propionate production was affected (P ≤ 0.05) by both the treatment and the bird age (Table 4). In addition to primary VFAs, valerate and iso-valerate production was also observed, at various stages of growth.

**Table 4:** Volatile acid production in the cecum in BMD-fed and no BMD-fed group birds at different ages.

Days	Treatments	Acetate	Butyrate	Propionate
**0**	Control	0	0	0
**7**	Control	81.83	15.91	2.25
	BMD	81.83	15.91	2.25
**14**	Control	74.51	19.42	4.68
	BMD	76.52	15.86	4.23
**22**	Control	73.84	19.44	5.98
	BMD	75.07	21.35	3.16
**35**	Control	75.22	20.08	4.67
	BMD	76.19	17.92	5.88
**42**	Control	78.19	11.73	9.62
	BMD	76.32	16.99	6.68
**Treatment**	Control	77.18	17.41	7.26
	BMD	78.46	17.41	4.93
**Age**	**0**	0	0	0
	**7**	81.83443	15.9183	4.494529
	**14**	75.51668	17.64134	5.087745
	**22**	74.4525	20.64581	4.570078
	**35**	75.70764	19.00126	7.05479
	**42**	77.25501	14.36104	8.153614
SEM^**@**^		1.19	1.01	0.67
**P- value**				
**Treatment**		0.66	0.81	0.01
**Day**		0.0001	0.0003	0.01
Trt*Day^**#**^		0.81	0.02	0.8

^@^SEM: standard error of mean (n = 4)

^#^The * denotes interaction between the two parameters treatment and day

### Sequencing information

**Fig 1:**
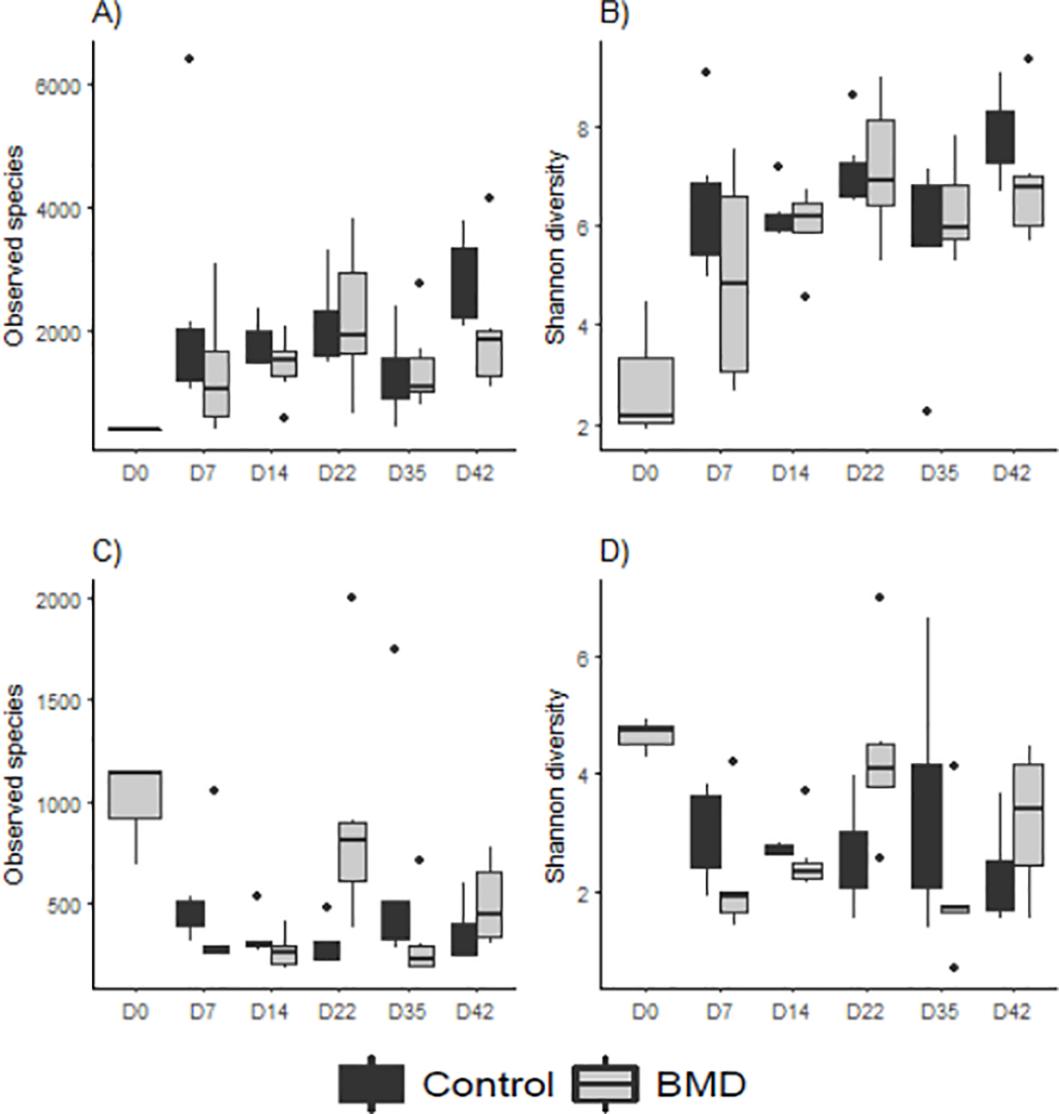
**Changes in microbial community species diversity and OTUs in ceca (A, B) and ilea (C, D).** Boxes represent the interquartile range (IQR) between the first and third quartiles (25^th^ and 75^th^ percentiles, respectively) and the horizontal line inside the box defines the media. Whiskers represent the lowest and highest values within 1.5 times the IQR from the first and third quartiles, respectively.

A total of 2,335,920 sequences were obtained after quality filtering, and 7, 28,555; 6, 95,798; 5, 17,383 and 3, 94,184 sequences were assigned to the cecum, ileum, litter and feed, respectively. A total of 55,192 OTUs were identified among the 41 samples of different groups examined for the cecum, ileum, feed and litter (S1 Table). The OTUs identified in the ileum are much lower than the OTUs identified in the cecum, and the overall number of OTUs increased as the bird advances in age. Furthermore, Good’s coverage for all samples was ≥ 0.90, indicating that the sequencing depth was sufficient for reliable analysis of these intestinal microbial communities (S1 Table). This is also reflected by the observed species and Shannon diversity index (Fig 1A–1D). The bacterial communities in the cecum were more diverse and rich compared to the ileal communities, and they shifted in parallel to one another as the chicks matured over time (Table 5). Interestingly, diversity and species richness was similar (P > 0.05) for the BMD-fed and control birds.

**Table 5:** Permutational ANOVA results partitioning effect of bird age, treatments and diet on microbial community composition of the cecum and ileum as calculated at a 97% cutoff as described in the text.

	Degree of freedom	Sum of Squares	F	Pr (>F)
Cecum vs. Ileum	1	4.18	69.75	<0.0001
***Weighted Unifrac Distance***				
In Cecum				
Diet	3	1.73	9.75	<0.0001
Treatment	1	0.11	1.93	<0.0001
Age	2	0.53	4.49	<0.0001
Age:Treatment	4	0.17	0.74	0.8596
In Ileum				
Diet	3	0.25	3.78	<0.0200
Treatment	1	0.02	0.75	0.2872
Age	2	0.11	2.51	<0.0020
Age:Treatment	4	0.15	1.62	0.0554
***Un-weighted Unifrac Distance***				
Cecum vs. Ileum	1	5.01	16.35	<0.0001
In Cecum				
Diet	3	2.43	2.91	<0.0001
Treatment	1	0.34	1.22	<0.0001
Age	2	1.05	1.89	<0.0001
Age: Treatment	4	1.19	1.06	0.2401
In Ileum				
Diet	3	1.45	1.77	<0.0004
Treatment	1	0.35	1.28	<0.0001
Age	2	0.97	1.78	<0.0001
Age: Treatment	4	1.47	1.34	<0.0002

### Influence of age, diet and antibiotic withdrawal on intestinal community composition

**Fig 2:**
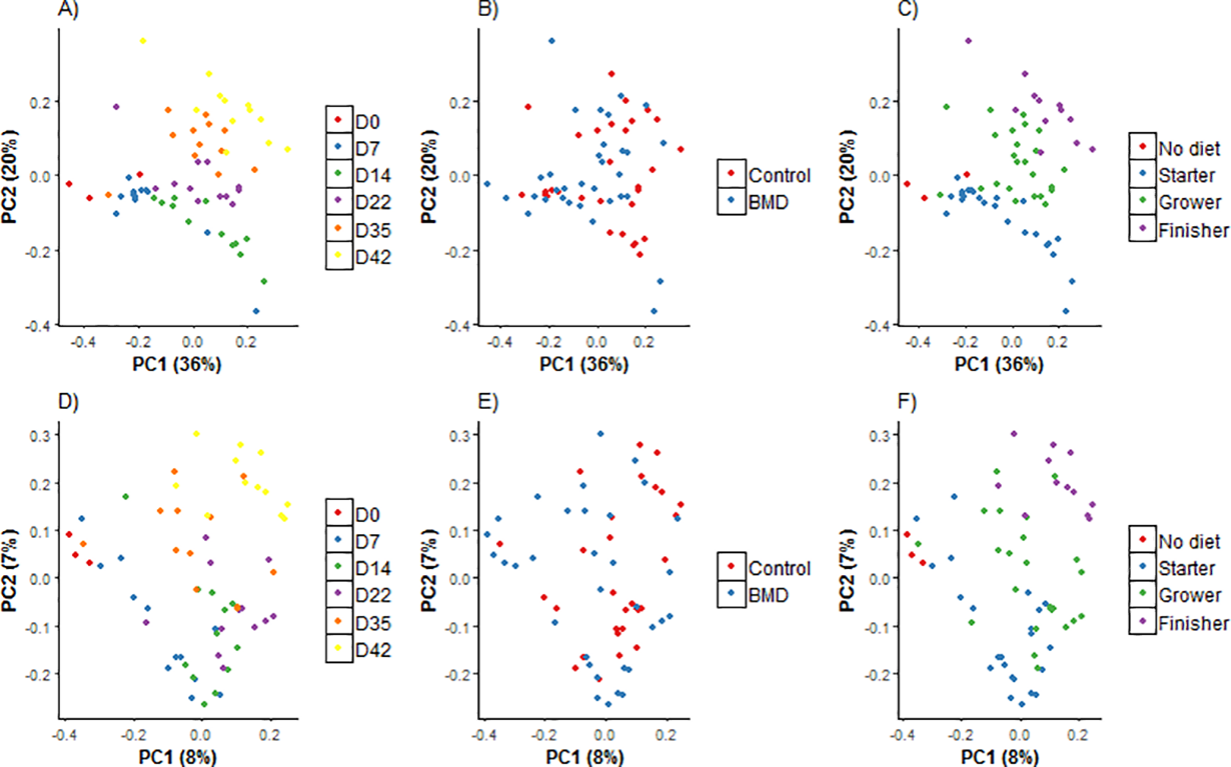
**Principal coordinate analysis (PCoA) shows weighted (based on abundance; A-C) and unweighted (based on presence/ absence; D-F) pairwise UniFrac distances between samples of cecum microbial community with respect of age (d0, 7, 14, 22, 35 and 42), treatment (BMD-supplemented and BMD-free (control) and diet (no diet: d0 birds fed no diet, starter (fed from d0-22), grower (d23-d35) and finisher (d35-42)**.

**Fig 3:**
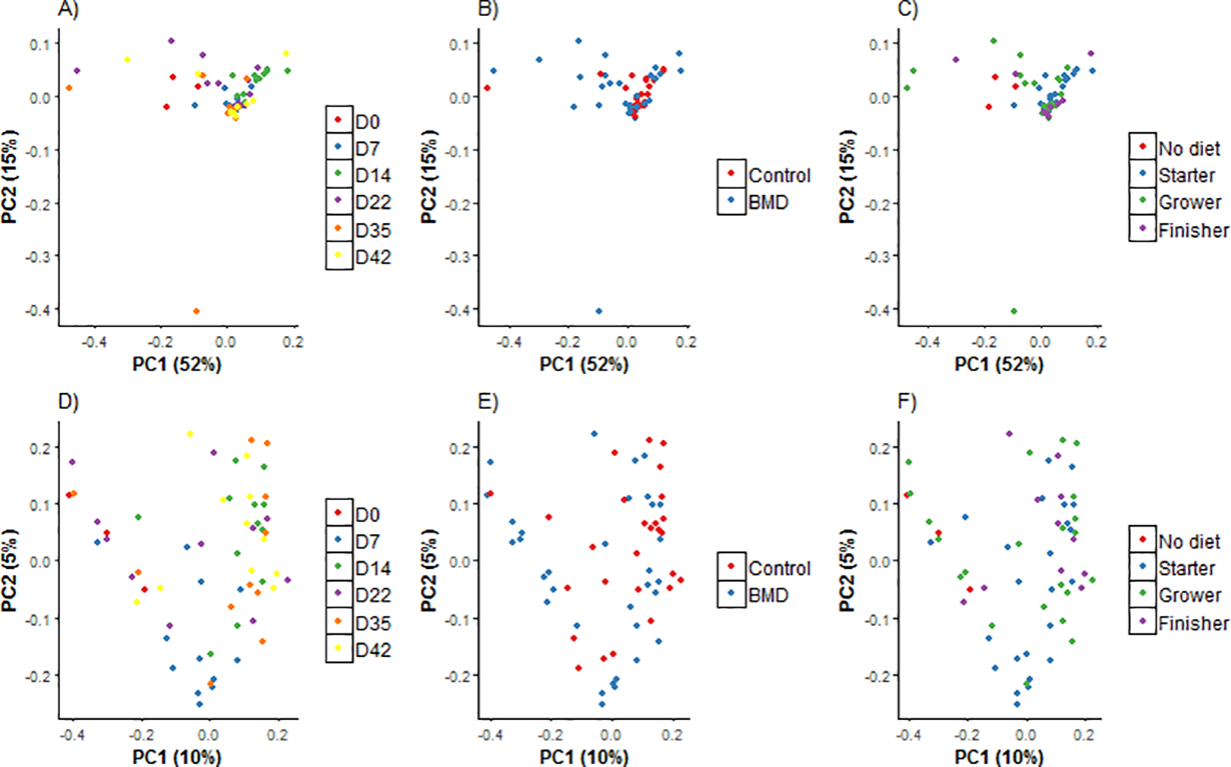
**Principal coordinate analysis (PCoA) based show weighted (based on abundance; A-C) and unweighted (based on presence/ absence; D-F) pairwise UniFrac distances between samples of ileum microbial community with respect of age (d0, 7, 14, 22, 35 and 42), treatment (BMD-supplemented and BMD-free (control) and diet (no diet: d0 birds fed no diet, starter (fed from d0-22), grower (d23-d35) and finisher (d35-42)**.

Community-level similarities of intestinal communities were analyzed using principal coordinate analysis (PCoA; Figs 2A–2F and 3A–3F) and are presented as a function of age, diet, and treatment [with antibiotic and antibiotic withdrawal (control)] and interaction between age: treatment. The cecal microbial community was more diverse (P ≤ 0.001) than that of the ileal microbial community. The composition of cecal bacterial communities was different (P ≤ 0.001; Permanova Table 5) at various stages of growth and in response to diets and treatments. Parallel to the cecal communities, ileal bacterial communities also showed the difference (P ≤ 0.05; Fig 3A–3C) at different ages and diet however, ileal community remained unchanged in response to antibiotic withdrawal.

### Taxonomic composition of bacterial community

**Fig 4:**
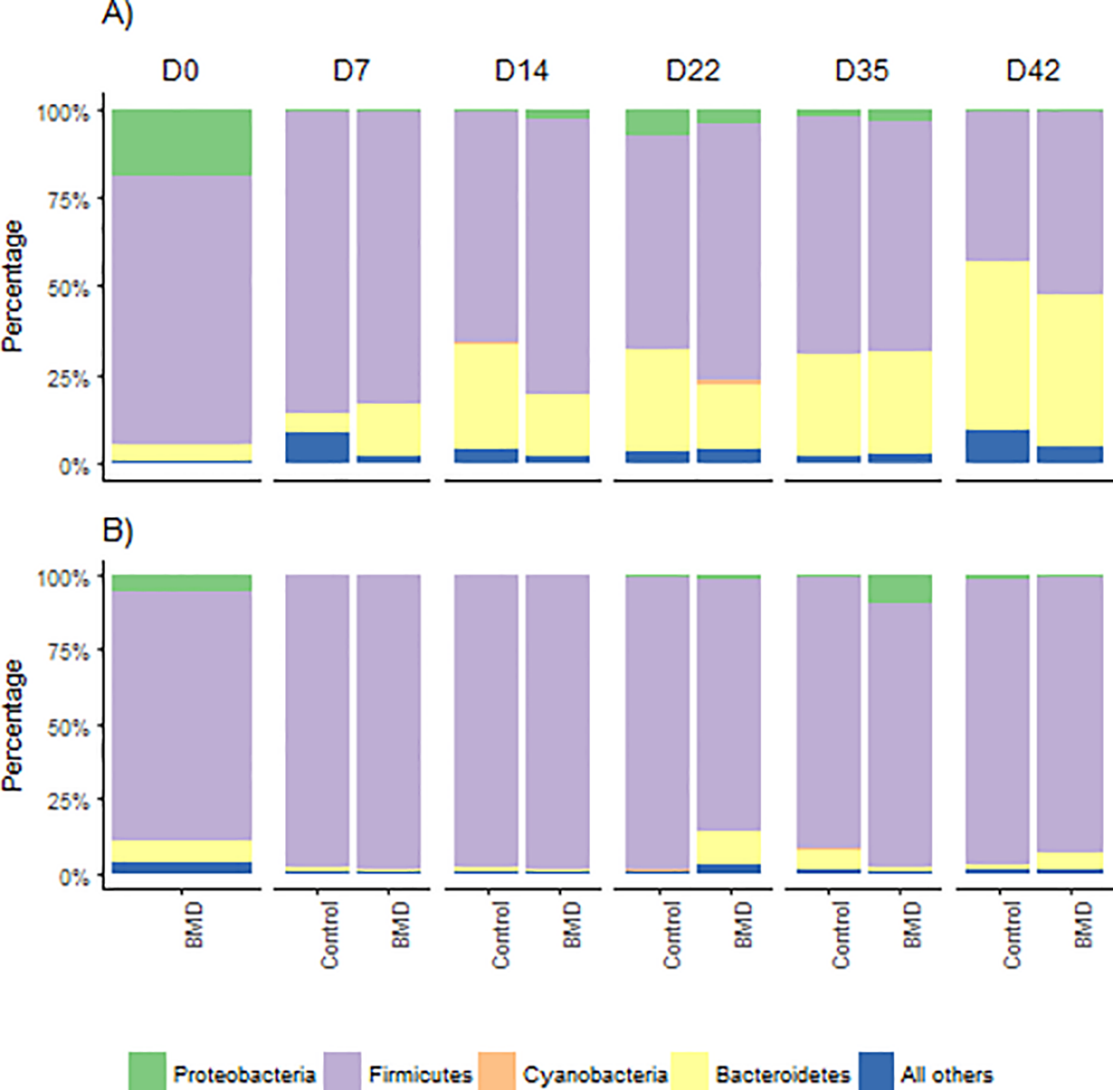
**Phylogenetic composition of (A) cecal microbial community and (B) ileal microbial community fed on BMD-diet and BMD-free diet (control group) across all ages (d0, 7, 14, 22, 35 and 42)**.

Phylum Firmicutes was the most abundant (49–90%) in both ilea and ceca of chickens at all ages and in both the groups, except d42 in the ceca (Fig 4A and 4B). Bacteroidetes was low in percent abundance (≤ 5.0%) at d0 in the ceca, however the percent abundance increased as birds advance in age, regardless of the antibiotic in the diet. Bacteroidetes was consistently found to be second most abundant phyla in the ceca. In contrast, Proteobacteria showed higher abundance (18.8%) at d0 but percent abundance decreased as the birds progressed in age.

**Fig 5:**
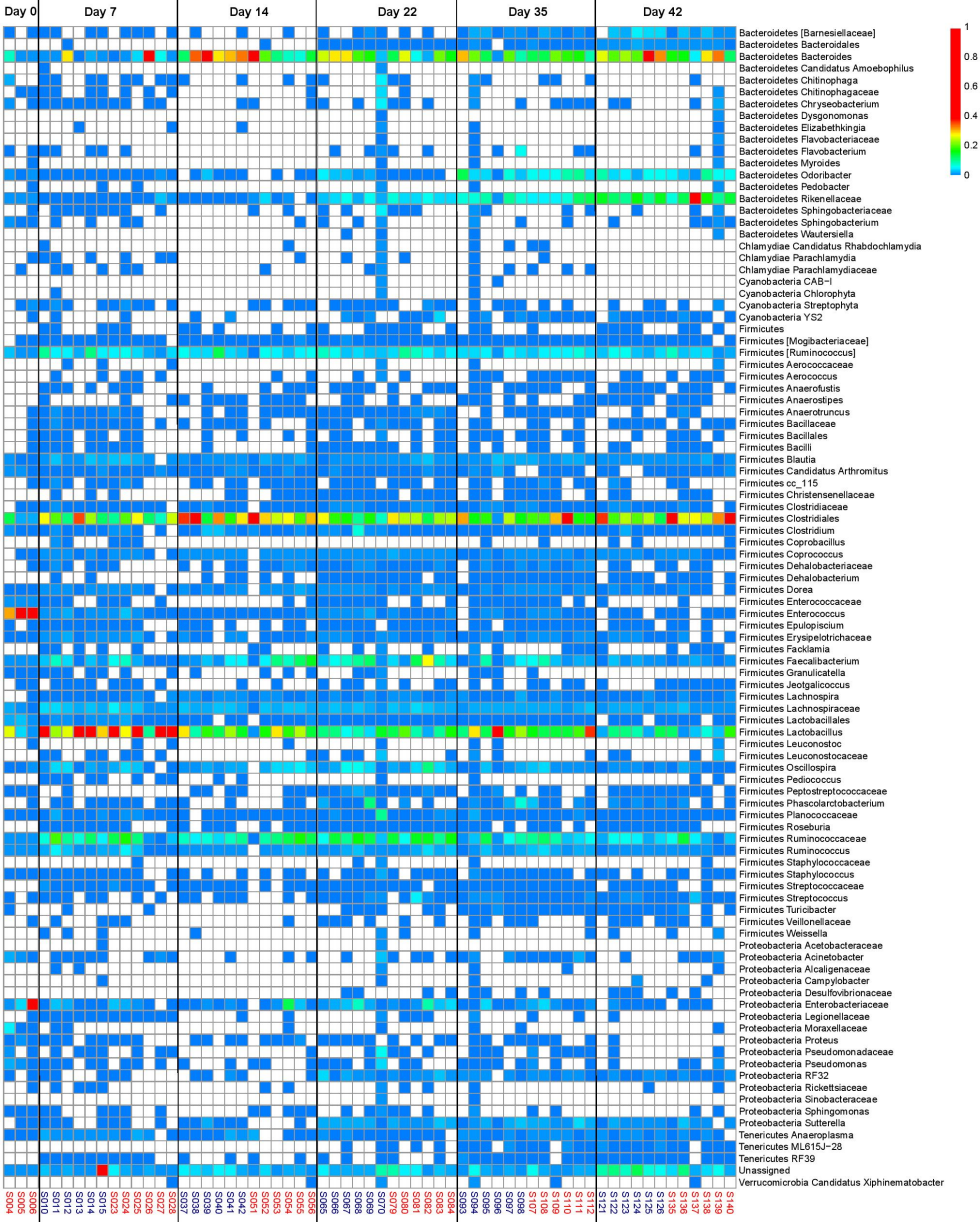
Heatmap of taxonomic groups present in the cecum of birds fed on BMD-diet and BMD-free diet (control group) across all ages (d0, 7, 14, 22, 35 and 42). The sample numbers (n = 6 for each time point) in red are of control diet and blue are of BMD diet.

**Fig 6:**
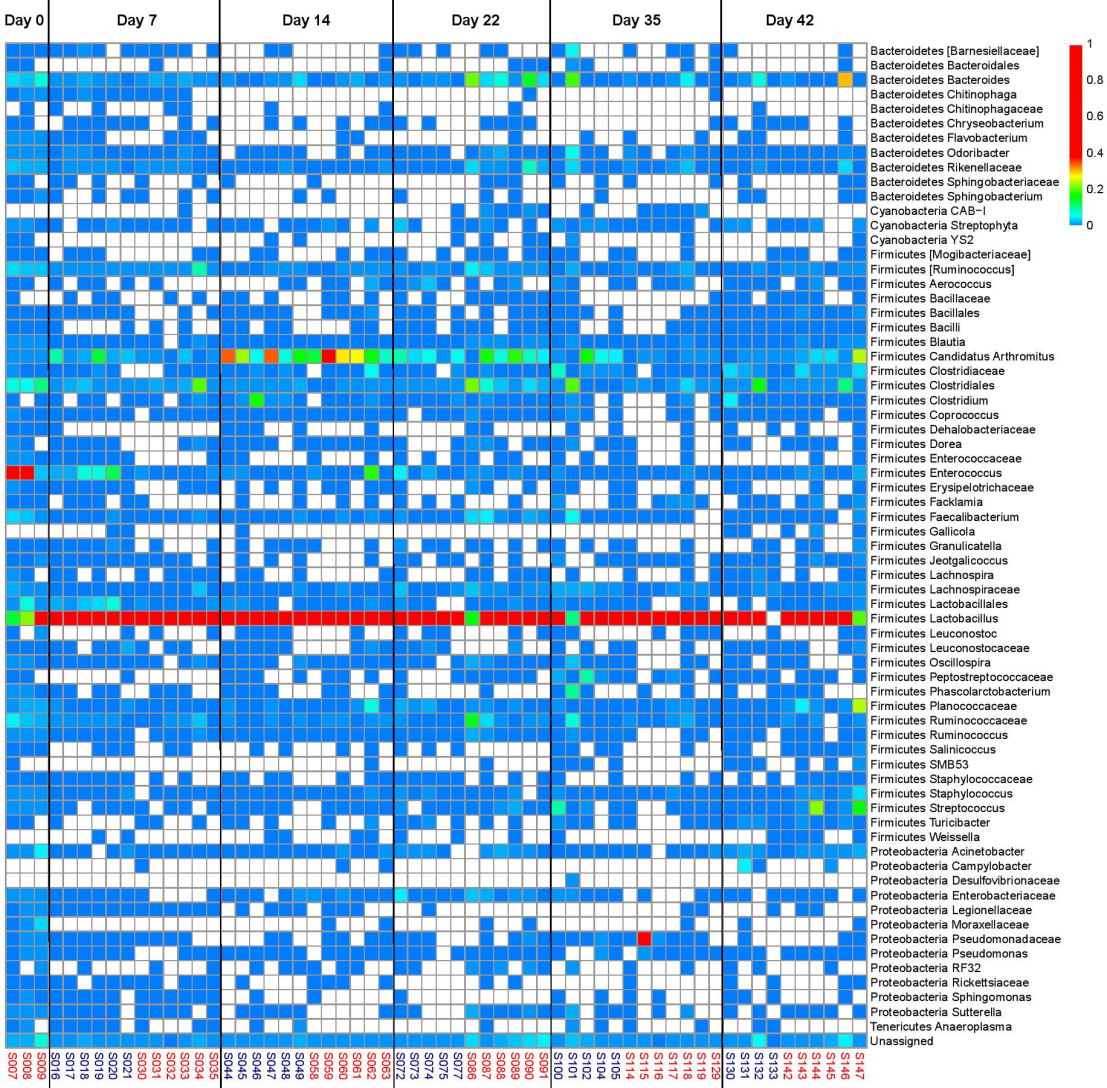
Heatmap of taxonomic groups present in the ileum of birds fed on BMD-diet and BMD-free diet (control group) across all ages (d0, 7, 14, 22, 35 and 42). The sample numbers (n = 6 for each time point) in red are of control diet and blue are of BMD diet.

*Bacteroides* was high in abundance, in the phylum Bacteroidetes, beyond d7 and a difference in abundance was observed between control and treatment (BMD-fed) groups at d14 (Fig 5). *Odoribacter* (beyond d35) and member of family *Rikenellaceae* showed higher abundance as the bird aged (beyond d22). Among Firmicutes, members of *Clostridiales*, and *Ruminococcus* were most dominant in the ceca irrespective of age, diet and antibiotic withdrawal (Fig 4). Genus *Lactobacillus* was highly abundant at d7, with lower abundance after d7. In contrast to the ceca, genus *Lactobacillus* was abundant in the ileum despite age, diet and antibiotic withdrawal (Fig 6). Candidatus division Arthomitus showed higher abundance in ilea particularly between d14-22. *Clostridiales* were abundant at d0 (≤ 0.2) and then their abundance decreased, however at d22, their abundance was higher in control group compared to BMD-fed group. Similar observation was made for genus *Bacteroides*. Day, diet and treatment interaction (P ≤ 0.0001) was significant for all the taxa under Firmicutes, except for order Lactobaccillales in ceca and ilea (S2 Table). Pathogenic bacteria *Campylobacter* was randomly identified in ceca and ilea during growth, regardless of antibiotic treatment.

Cecum, ileum, litter and feed samples were also examined at the OTU level to find specific subsets of OTUs which represents each sample type (S1 Fig). Analysis clearly indicated that there were shared subsets of OTUs between sample types, and also unique subsets of OTUs that were particular to each sample type. For instance, OTUs representing members of family *Veillonellaceae* and genus *Roseburia*, *Anerofustis* and *Corpobacillus* were unique to cecum samples (Fig 6). OTUs representing Firmicutes SMB53 was detected only in ileum, while genus *Weissella and Jeotgallicoccus* were detected across all the samples (S2 and S3 Figs). OTU representing Candidatus division Arthomitus, a segmented filamentous bacteria (SFB), was only detected in ileum and cecum samples. Further, our analysis also showed that large subsets of OTUs were shared between litter and cecum.

### Expression pattern of cytokine genes

**Fig 7:**
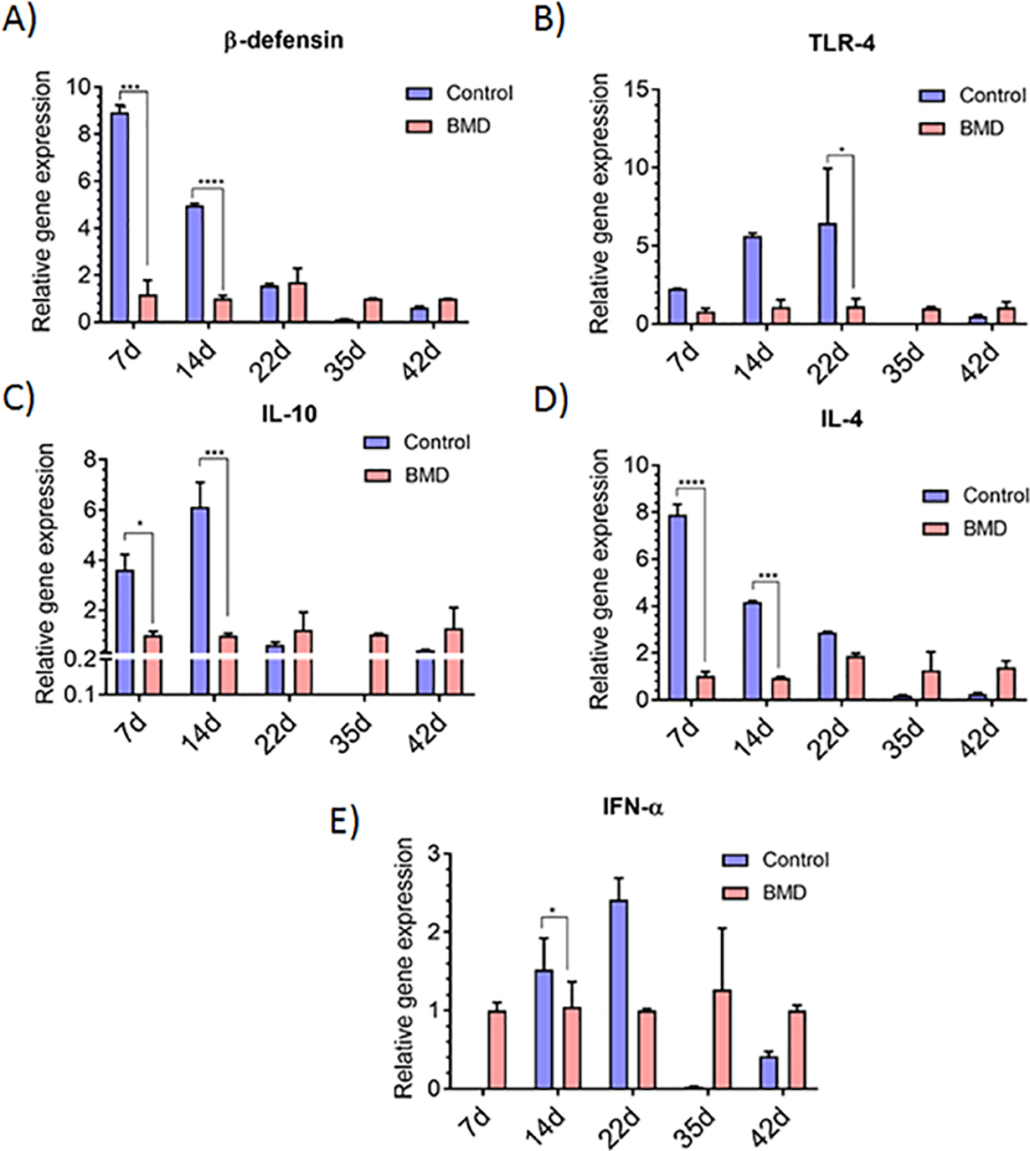
**Change in cytokine gene expression (A) β-defensin (B) TLR-4 (C) IL-10 (D) IL-4 and (E) IFN-α in the ceca.** Values are represented as means ± standard error of mean, where n = 4.

**Fig 8:**
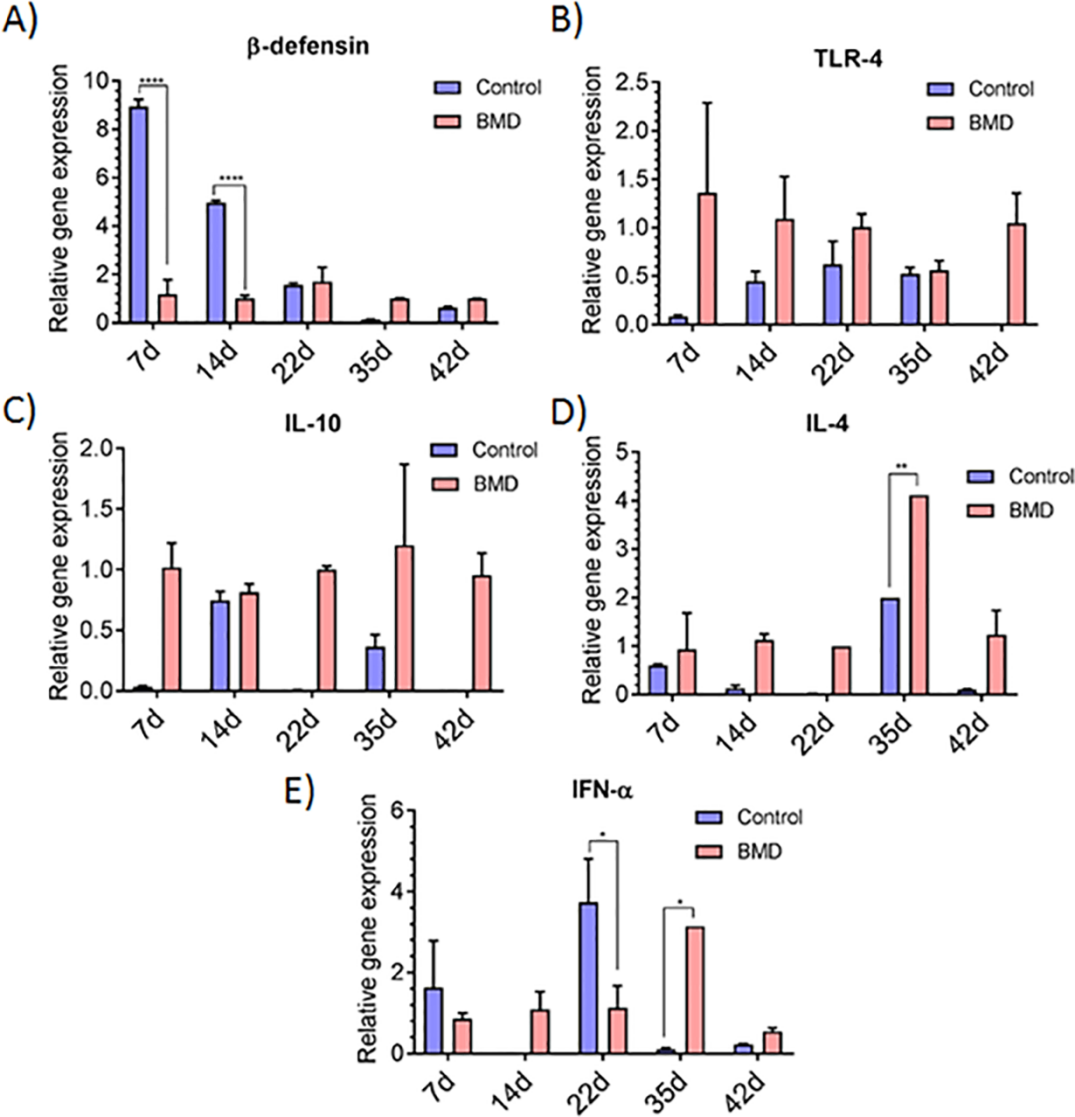
**Change in cytokine gene expression (A) β-defensin (B) TLR-4 (C) IL-10 (D) IL-4 and (E) IFN-α in the ilea.** Values are represented as means ± standard error of mean, where n = 4.

Besides evaluating the effect of antibiotic withdrawal on bird performance and microbial community, we also evaluated its effect on cytokine genes (IL-10, IL-4, IFN-γ, beta-defensin, and TLR-4) expression of the host. Expression for beta-defensin and Il-4 gene was higher (P ≤ 0.05) at d7 in the BMD-fed group and decreased gradually with increasing age of bird, in the ceca (Figs 7A–7E). The expression for IL-10, TLR-4, and IL-4 genes were different (P ≤ 0.05) for the control and treated groups at initial stages of growth. The expression for IFN- γ, IL-10, and TLR-4 increased gradually (P ≤ 0.05) from d7 to d14 or d22 (in case of IFN- γ) and then decreased sharply at d35. Similar to the ceca, gene expression was different (P ≤ 0.05) in the ilea of BMD-fed and control groups except IFN- γ. However, trends in gene expression pattern were not observed in the ilea with respect to bird age (Fig 8A–8E) as observed in the ceca.

### Abundance of enteric pathogens

**Fig 9:**
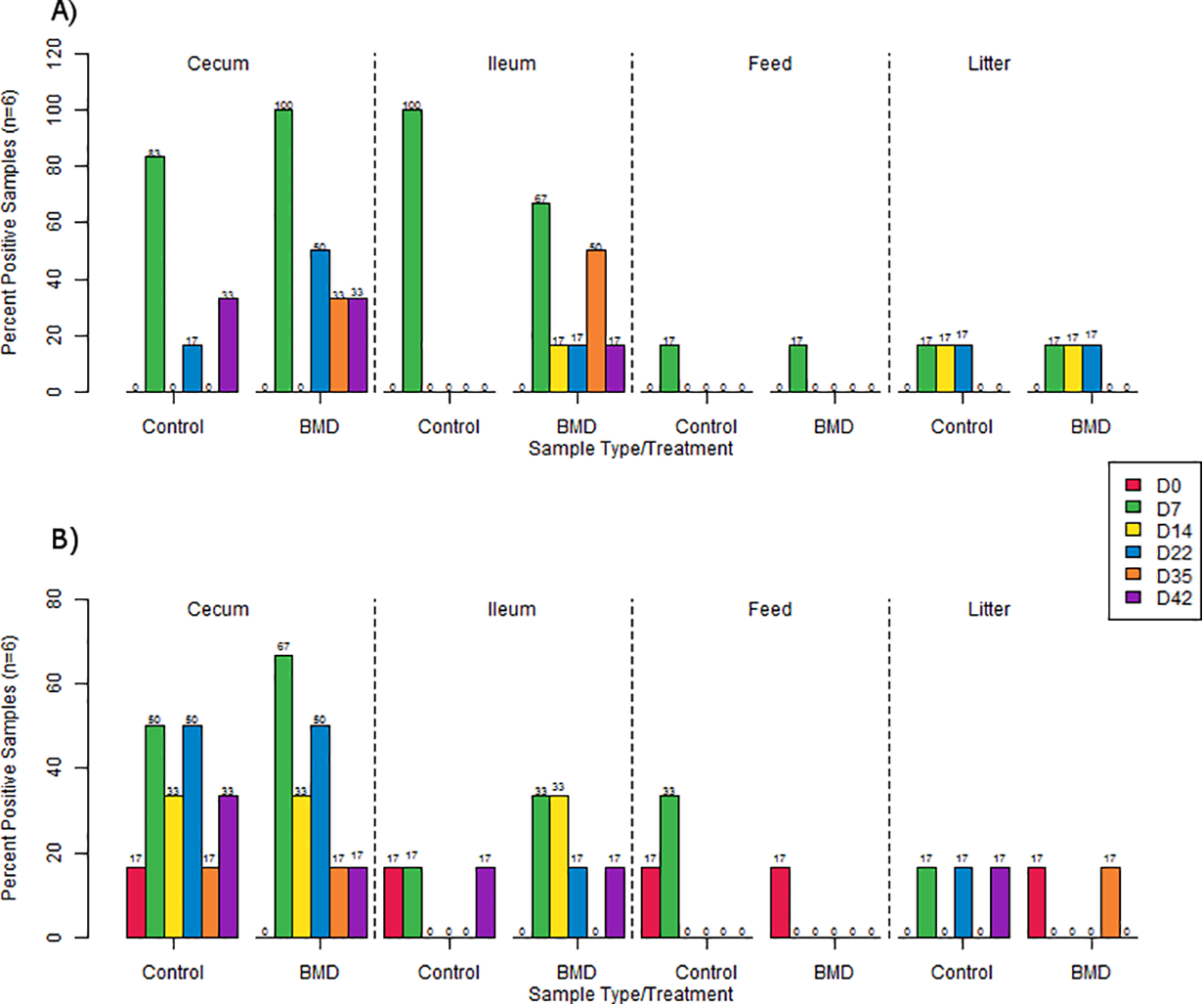
Prevalence of food borne pathogens in cecum, ileum, litter and feed collected at different time points (d0, 7, 14, 22, 35, 42) from BMD-free and BMD supplemented groups. (A) Prevalence (in %) of *Salmonella* and (B) Prevalence (in %) of *Campylobacter*.

At d0, *Salmonella* spp. were not isolated from the cecum or the ileum contents however, as bird aged, prevalence of *Salmonella* spp. increased during the early growth stage and their abundance decreased with increasing bird age, regardless of treatment. *Salmonella* spp. prevalence in feed and litter samples was 25.0 (3/12) and 41.7% (5/12), respectively. In contrast, *Campylobacter* spp. were detected in all the samples (cecum, ileum, feed and litter) at d0. Cecum showed higher prevalence of *Campylobacter* spp. irrespective of age and antibiotic, while both *Campylobacter* spp. and *Salmonella* spp. showed higher prevalence in ilea of BMD-fed chicks compared to control group (Fig 9A and 9B).

## Discussion

The purpose of this study was to examine the effect of antibiotic withdrawal from feed on bird performance, host immunity and microbial community in the cecum and ileum over the time. Concurrently, prevalence of foodborne pathogens in the ceca and ilea was also investigated. Similar to earlier published reports [3, 5, 30, 31], our study observed an increase in production performance response to dietary supplementation of bacitracin in the early phase (until d22) of chick growth. However, growth performance response to in-feed antibiotics was inconsistent across all ages [16] and reports indicate growth depression in broilers fed salinomycin [32]. In addition, it is well-documented that changes in the microbial community at strain level [16] can have implications on bird performance. Since supplementation of antibiotics can alter gut microbial community, strain-specific physiognomics and/or host selective pressures determining the process of colonization could influence performance outcomes.

VFAs are the major end metabolites for gut microbial fermentation and its efficient production is important to the bird's health as it not only meets the energy demands but also acts as a carbon source [7, 33, 34]. It is well-established that oligosaccharides supplementation increases fermentation in the ceca, producing volatile fatty acids, primarily acetate, propionate and butyrate [7, 31, 35, 36]. In addition, small quantities of valerate and iso-valerate were also detected [34], as observed in our study. The fermentation of the ingested feed increases as the bird mature and the bacterial community gets established [7, 37].

Gastrointestinal tract harbors diverse and complex microbial community and therefore, extensive interactions occur between the microbial cells and host immune system. Beta-defensins are small antimicrobial peptides that are present on the intestinal epithelial surface and constitute an important part of innate immunity [7, 38]. They are induced in response to challenge by lipopolysaccharide, involving Toll-like receptors (TLR-4) and the transcription factors NFκB [39]. Expression of beta-defensin in the first week of age in the gut was reported in literature [40, 41], as observed in this study. Since beta-defensin produced in heterophils, major components in the chick’s arsenal against pathogens, their increased expression in response to *Salmonella* infection was reported by Brisbin et al. [42, 43]. In our study, *Salmonella* and *Campylobacter* were detected in the chick’s gut at an early growth period. Therefore, the higher expression of beta-defensin and TLR-4 is reasonable. The innate immune response leads to subsequent adaptive immune response and stimulates the production of Th2 cytokines (IL-4 and IL-10) [43]. In our study, we observed increased expression of IL-4 and IL-10 in the early growth phase of the bird. Expression of the IL-4 and IL-10 decreased at later stages of growth. These changes in the host gene expression can be attributed to modulation in the microbial community due to age, antibiotic administration and pathogen infection. Antibody-mediated immune response due to modulation in gut microbiome through the administration of antibiotics and probiotics has been reported [44, 45]. In addition to the antibody-mediated immune response, gut microflora also affected the cell-mediated immune response (IFN-γ) [46]. In this study, we observed an increase expression of IFN-γ till d22, and this can be ascribed to changes in the bacterial community parallel to the age of the bird. Oakley and Kogut, [46] also reported temporal changes in the pro-inflammatory cytokine expression in the chicken gut and their report corroborates with our results.

Correlations of the relative abundance of bacterial taxa with cytokine gene expression revealed that Proteobacteria are correlated with anti-inflammatory and pro-inflammatory responses (IL-6, IL-10), whereas the abundance of Firmicutes is inversely proportional [46]. In our study, we observed greater abundance of Proteobacteria during early stage of growth (d0-7) which was later replaced by Firmicutes as the bird aged. These results indicate that modulation in commensal bacteria as the bird matures may also affect the host immunity. However, greater statistical power (using higher pen and bird numbers or at a commercial facility) is needed to ascertain the immunomodulatory effect of commensal gut flora.

Earlier reports on the poultry gut microbiome were based on data obtained through the conventional microbiological approaches [47, 48] and/or early molecular fingerprinting methods [34, 49–55]. However, there are only a few HT-NGS based reports on taxonomical differences and development of the microbial community in chicken gut. In the recent past, Danzeisen et al. [2] investigated the effect of anti-coccoidial monensin and growth promoter treatments on the chicken cecal microbial community, while Stanley et al. [55] correlated the bacterial community with the efficiency of extraction from the feed. However, these studies targeted different hypervariable (V3 and V1-V3) regions of 16S rRNA for sequencing. In the present study, we targeted V3-V4 region along with longer MiSeq read chemistry for a better resolution in microbial diversity and OTUs classification. In accordance with an earlier report [56], ceca have greater species richness and diversity compared to ilea. And this complexity and diversity increases as the bird ages [3, 13, 14, 56, 57]. It has been well-documented that the small intestine including the ileum is the main location for nutrient absorption; whereas most of the fermentation takes places in the ceca [56]. Moreover, diet had a significant effect on gut microbial communities. Small changes in dietary cereal grain (carbohydrate source) composition can remarkably affect the intestinal bacteria at strain level [58]. Not only grain, but source and level of protein may lead to increase or decrease of certain bacterial population. Sun et al. [58] observed an increase in lactobacilli and a decrease in coliform population when soybean meal was replaced with fermented cottonseed meal as a protein source. Since we had three different diets [starter (d0-22), grower (d23-35) and finisher (d36-42)], changes in the gut microbial community over time can be confounded with the change in diet. However, no significant effect of antibiotic treatment was observed in the gut community.

Firmicutes constituted the most predominant phylum (average of > 70%) in both ceca and ilea during bird growth. However, at d42, their abundance declined (< 50%) in the ceca. These findings are in agreement with earlier reports [2, 59–62]. The decrease in Firmicutes and increase in Bacteroidetes at d42 is due to shift from starter diet (corn starch: 53.38%; CP: 23.0% and oil: 5.60%) to finisher diet (corn starch: 69.59%; CP: 18.0% and oil: 3.09%). Bacteroidetes play an important role in converting fermentable starch to simple sugars and eventually, volatile fatty acids to meet the energy demand of the host [63]. Bacteroides, Clostridiales and Ruminococcus were dominant in the cecum, while *Lactobacillus* was predominant in the ileum. The dominance of *Clostridia* and *Bacteroidia* in the cecum was also reported by Danzeisen et al. [2]. Similar to our study, Shaufi et al. [56] reported a higher abundance of *Clostridia* at d7 and 21 in the ilea. However, these finding contradicts with Lu et al. [63] who reported dominance of *Clostridia* in both ilea and ceca. These differences in taxonomic composition can be expected due to differences in the approaches. Compared to our MiSeq approach, Lu et al. [63] used molecular fingerprinting technique. In addition, DNA extraction methods, different primer pairs, environmental factors, treatment, dietary composition, feed additives, chicken breed, and climate may pose a challenge when comparing the taxonomic groups and OTUs from different studies [56, 64, 65]. The abundance of *Lactobacillus* in the ilea is in accordance with earlier reports [55, 64, 66, 67]. On the contrary, Shaufi et al. [56] showed ≤ 4.0% abundance of *Lactobacillus* in the ileum.

Furthermore, we observed the difference in abundance of Clostridiales and genus *Bacteroides* in treated and control groups, however, this difference was not observed across all ages. The exact reasons for this difference is unclear but could represent an increase and/ or decrease of other unidentified bacteria, in the course of establishment of bacterial community, and occupation of an available niche within the gut resulting in an overall balance of Firmicutes and Bacteroidetes. As evident from this study, interactions between bird age and diet influenced the microbiome than did treatment effects, similar to the work of Danzeisen et al. [2] and Ballou et al. [13]. Though the effect of antibiotic was not observed in the bacterial community at the higher taxonomical level, a significant difference at lower taxonomic level can be observed over the time.

The differences in OTUs between the cecum, ileum, and litter samples were largely within the phylum Firmicutes. However, at later stages of growth, members of phylum Bacteroidetes were shared between feed, litter and cecum samples. Genus *Weissella*, which is reported to be in high abundance in healthy birds, was detected in our gut and feed samples [3, 14]. Lactic acid bacteria, *Jeotgallicoccus* was detected across all the samples and contradicts to earlier reports of Danzeisen et al. [3, 14] who showed its presence only in litter and ileum. The presence of Candidatus division Arthomitus was reported only in ileum samples by Danzeisen et al. [3, 14]. However, in our study, SFB was detected in both ileum and cecum samples. SFB are commensal bacteria and were first identified in the ilea of mice and rats and its colonization in the gut influences the host innate immune system [68]. Poultry litter harbors a complex microbial community and have a potential effect on the intestinal microbial community [3, 7]. In this study, we used fresh litter and it was mainly dominated by *Lactobacillus* spp. Similar to the study of Cressman et al. [15], the ileal microbial community was also dominated by *Lactobacillus* spp., indicating the correlation between litter and ileal samples in the current study [3].

Species of *Campylobacter* and *Salmonella* are important foodborne pathogens commonly associated with poultry and poultry products. These pathogens not only cause economic losses via poor bird performance but also leads to serious infections in the human when consumed [62, 69–71]. It has been reported that litter status and dietary antibiotics are key factors that can influence enteric pathogens [62]. In this study, we compared the prevalence of *Salmonella* and *Campylobacter* in the ileum, cecum, feed and litter samples of BMD-fed and control groups. Our study showed a high level of *Campylobacter* and *Salmonella* in the ilea of BMD-fed chicks which is congruent with the study of Wei et al. [62] who also reported a high level of *Salmonella* spp. in ileal mucosa of chicks despite bacitracin treatment. The prevalence of *Salmonella* and *Campylobacter* in chick’s fed-bacitracin can be attributed to its activity against Gram-positive bacteria. Inhibition of Gram-positive bacteria can lead to the proliferation of Gram-negative bacteria, including *Salmonella* and *Campylobacter*, as competition for nutrients is reduced.

In summary, the present study identified that the cecal microbial community is affected by diet, age and antibiotic treatment compared to ileum which was affected only by diet and age. At early stages of growth, bird performance and host immunity are significantly affected by the antibiotic supplementation in the diet. Inclusion of antibiotic in diet had resulted in the higher prevalence of foodborne pathogens. Considering the descriptive nature of these results, it is difficult to define the beneficial role of modulated bacterial community in response to antibiotic supplementation during growth. However, due to the controlled and hygienic environment of our research facility, microbes encountered in the environment are probably not much diverse than those encountered in commercial poultry production units. Furthermore “Omic” approaches are needed to gain in-depth knowledge of functions associated with the modulated gut microbial community as well as newly identified bacteria. This study provides a more inclusive view on the effect of antibiotics on the poultry gut microbial modulations, bird performance, host immunity and pathogen prevalence, might help in designing alternative strategies for replacing antibiotics in modern poultry production and for assuring food safety.

## Supporting information

S1 Table Number of OTUs per groups and estimators of sequence diversity and richness. (XLSX)

S2 Table Mean sample (%) of bacterial taxa (identified to the genus level) in BMD-fed and control chicken groups. (XLSX)

S1 Fig Venn diagram showing the number of shared OTUs in ceca and ilea with litter and feed. (TIFF)

S2 Fig Heatmap of taxonomic groups present in the litter of BMD-fed and control groups across ages. (TIFF)

S3 Fig Heatmap of taxonomic groups present in the feed BMD-fed and control group across ages. (TIFF)
