# Supplementary figures and images for: Effect of antibiotic withdrawal in feed on chicken gut microbial dynamics, immunity, growth performance and prevalence of foodborne pathogens
Source: PLoS One. 2018 Feb 14;13(2):e0192450. doi: 10.1371/journal.pone.0192450 (PMC5812630; doi:10.1371/journal.pone.0192450)

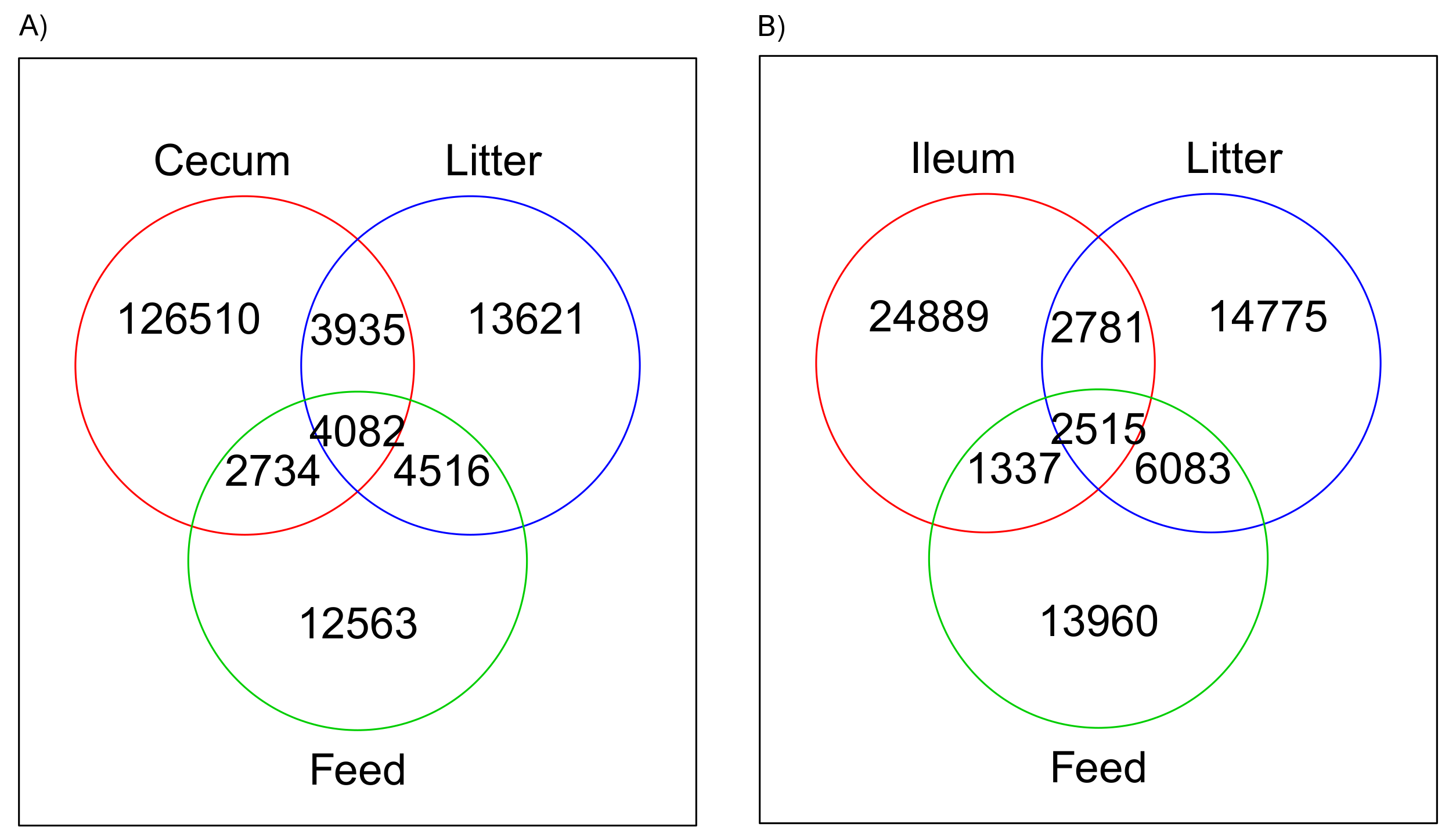

Supplement: S1 Fig — (TIFF) [file pone.0192450.s003.tiff]

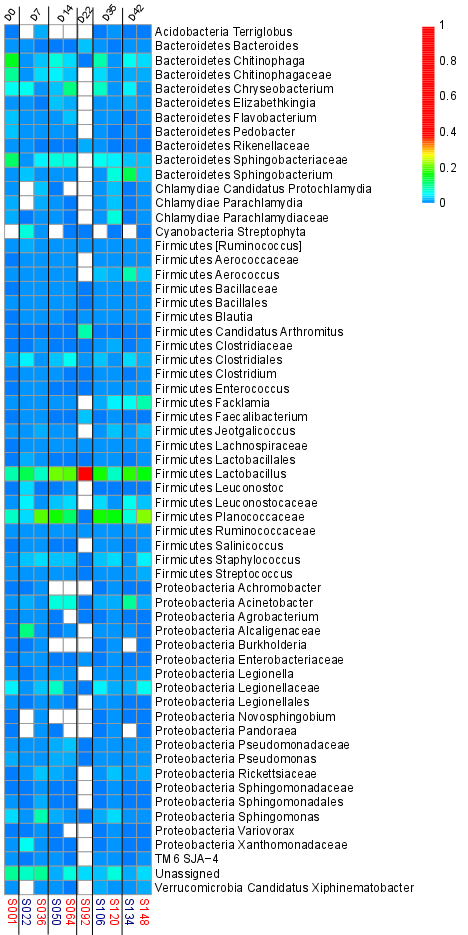

Supplement: S2 Fig — (TIFF) [file pone.0192450.s004.tiff]

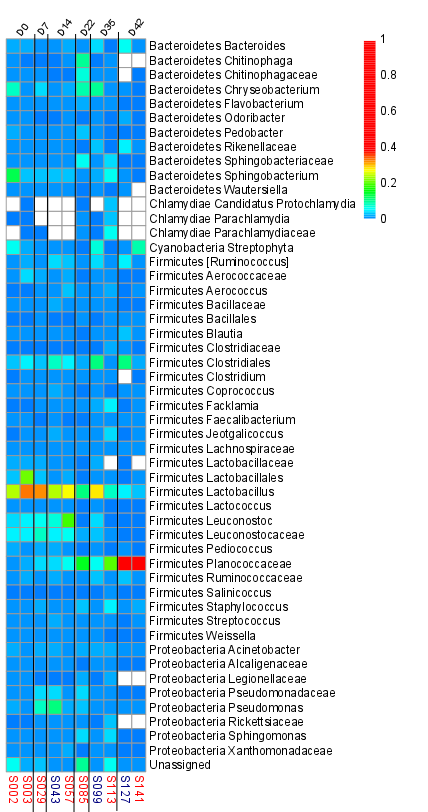

Supplement: S3 Fig — (TIFF) [file pone.0192450.s005.tiff]
